# Cholesterol Lowering Effect of Plant Stanol Ester Yoghurt Drinks with Added Camelina Oil

**DOI:** 10.1155/2016/5349389

**Published:** 2016-02-21

**Authors:** Pia Salo, Päivi Kuusisto

**Affiliations:** ^1^Research Centre of Applied and Preventive Cardiovascular Medicine, University of Turku, Kiinamyllynkatu 10, 20520 Turku, Finland; ^2^Raisio Nutrition Ltd., P.O. Box 101, 21201 Raisio, Finland

## Abstract

The aim of this study was to investigate the effects of yoghurt minidrinks containing two doses of plant stanol ester either with or without added camelina oil on the serum cholesterol levels in moderately hypercholesterolemic subjects. In this randomised, double-blind, parallel group study, 143 subjects consumed a 65 mL minidrink together with a meal daily for four weeks. The minidrink contained 1.6 or 2.0 grams of plant stanols with or without 2 grams of alpha-linolenic acid-rich camelina oil. The placebo minidrink did not contain plant stanols or camelina oil. All plant stanol treated groups showed statistically significant total, LDL, and non-HDL cholesterol lowering relative to baseline and relative to placebo. Compared to placebo, LDL cholesterol was lowered by 9.4% (*p* < 0.01) and 8.1% (*p* < 0.01) with 1.6 g and 2 g plant stanols, respectively. With addition of Camelina oil, 1.6 g plant stanols resulted in 11.0% (*p* < 0.01) and 2 g plant stanols in 8.4% (*p* < 0.01) reduction in LDL cholesterol compared to placebo. In conclusion, yoghurt minidrinks with plant stanol ester reduced serum LDL cholesterol significantly and addition of a small amount of camelina oil did not significantly enhance the cholesterol lowering effect. This trial was registered with ClinicalTrials.gov NCT02628990.

## 1. Introduction

Plant stanols are a subgroup of plant sterols. Both are naturally found, for example, in vegetable oils and cereals, and are therefore available in small amounts in everyday diets. By chemical structure, plant sterols and plant stanols resemble cholesterol but, when ingested, they are not absorbed to any significant degree from the gastrointestinal tract. Importantly, plant sterols and plant stanols have been shown to reduce absorption of cholesterol and, consequently, serum total and LDL cholesterol concentrations.

Diet is a cornerstone in the management of moderate dyslipidemia. Plant stanols and plant sterols are an acknowledged dietary means to effectively reduce elevated serum LDL cholesterol [1]. Numerous randomized, placebo-controlled clinical studies have consistently shown that serum total and LDL cholesterol concentrations are reduced effectively with regular daily use of plant sterols and plant stanols [2–6].

A significant cholesterol lowering effect was first demonstrated when plant stanol ester was added in vegetable oil based margarines and mayonnaises [7, 8]. Both are high-fat foods and typically consumed as part of a meal and as several daily doses. More recently, cholesterol lowering effect has been studied in different food matrices [4–6]. Today, probably the most commonly consumed plant stanol and plant sterol products worldwide are yoghurt minidrinks in which the daily recommended dose has been added in single serving of 65–100 mL. With plant sterol ester, LDL lowering effect has been studied with doses ranging from 1.6 g to 3 g/d plant sterols with variable results considering efficacy [9–12]. With plant stanol ester, minidrinks with 1.6 g plant stanols have not been previously studied, but only studies with plant stanol doses 2 g/d or higher in minidrinks exist [13–18].

Plant stanols reduce the absorption of both dietary and biliary cholesterol by interfering with micellar transport of cholesterol from the intestinal lumen to the enterocytes lining the intestinal wall. The mode of action of plant stanols and plant sterols needs to be taken into consideration when developing different types of foods as vehicles for plant stanols and plant sterols. Poor efficacy has been shown if foods containing plant sterol ester or plant stanol ester have been taken without a sufficient meal [9, 19]. This is understandable since the food products containing plant stanol ester or plant sterol ester must be consumed in such a way that when ingested, the food stimulates the release of (1) digestive enzymes to hydrolyse stanol/sterol ester into its active form, that is, plant stanols or sterols, and (2) digestion hormones (such as CCK) to release bile acids into the duodenum for micellar formation [20–22]. The necessary stimulus for these functions to occur is probably a large enough volume of food with high enough content of fat [23]. Products such as minidrinks and biscuits can easily be consumed as a quick snack, either alone or together with other snacks, and not as part of a meal. Ingested alone, their volume may be too small and the fat content too low to trigger the relevant digestive processes. Also the gastric emptying of a minidrink may be too fast, if ingested as such on an empty stomach [24]. Therefore, in most clinical trials subjects have been advised to consume test products with meals. Despite such advice, people may still use them as snacks [25].

Usually the plant stanol ester and plant sterol ester minidrinks are prepared of fat-free milk or contain only a small amount of dairy fat. However, because of the obvious challenges with the consumption pattern of minidrinks, addition of a small amount of fat in the minidrinks might promote the cholesterol lowering efficacy of the drinks especially if they are consumed without a meal, but also possibly when taken with meals. Doornbos et al. did not find a difference in the LDL lowering effect between two minidrinks containing plant sterol ester with low or slightly higher content of dairy fat (0.1 g or 1.5 g) when consumed without a meal or together with lunch. The fat content of the lunch did not have any significant effect either [9]. However, Nissinen et al. showed that even when taken together with a meal, the fat content of the meal affected the hydrolysis rate of plant stanol ester [20].

Studies with triglyceride emulsions have shown that emulsions with solid fat are digested more slowly in the stomach and the duodenum as compared to emulsions with more liquid fat [26, 27]. In the commercial plant stanol ester, plant stanols are esterified with rapeseed oil fatty acids. This type of plant stanol ester has a solid appearance at room temperature (20°C) and melts gradually when the temperature is raised in a similar way as vegetable fats, such as palm oil, or milk fat. However, the plant stanol ester is not totally liquid at body temperature. Typically the solid fat content (SFC) of plant stanol ester is 10–15% at 35°C and 5–8% at 40°C. Plant stanol ester has higher melting temperature as compared to plant sterol ester esterified with the same fatty acid blend. The commercial plant sterol ester, where plant sterols are esterified with sunflower oil fatty acids, usually contains less solid fat both at room temperature and at body temperature than the plant stanol ester and thus there may also be slight differences in the hydrolysis rates of these esters. Actually in the study of Lubinus et al. plant stanol esters tended to be hydrolysed to a lesser extent than plant sterol ester [28]. However, the products were not advised to be taken with meals, which may also have affected the results.

Keeping all of this in mind, we hypothesized that blending a liquid oil and plant stanol ester together before emulsification into a yoghurt drink may promote effective incorporation of the emulsified oil-plant stanol ester blend into the fat phase in the stomach and possibly enhance the hydrolysis rate of plant stanol ester and thereby enhance the cholesterol lowering efficacy of plant stanol ester in a minidrink-type product. Addition of a vegetable oil, with high content of polyunsaturated fatty acids, into plant stanol ester product is also of a further benefit since it helps to improve the overall fatty acid composition of the diet.

The aim of the current study was therefore to evaluate whether addition of a small amount of vegetable oil could improve the LDL lowering effect of plant stanol ester on serum lipids. Thus, we carried out a trial where we investigated the LDL lowering effect of two doses of plant stanols, 1.6 grams and 2.0 grams, with and without 2 grams of omega-3-rich camelina oil (*Camelina sativa*).

## 2. Subjects and Methods

### 2.1. Subjects

Subjects were recruited by an advertisement in a local newspaper and advertisements on websites of the local enterprises in close proximity of Faculty of Medicine at University of Turku. Subjects who were interested were first interviewed by phone. Those who met the inclusion criteria and none of the exclusion criteria received information about the purpose and the protocol of the study and were scheduled for a screening visit at the study unit. Inclusion criteria were age 25–65 years; normal weight or being slightly obese (BMI < 30 kg/m^2^); moderate hypercholesterolemia (fasting total cholesterol between 5 and 8 mmol/L); and triglycerides < 3 mmol/L. Exclusion criteria were regular use of food products containing plant stanols or plant sterols, hyperglycemia or diabetes, hyper- or hypothyroidism, current use of lipid-lowering, hypertension or other cardiac medications, atherosclerotic cardiovascular disease, malignancy, pregnancy, lactation, or alcohol abuse. On the basis of these criteria, 155 subjects were recruited.

### 2.2. Study Design

This was a randomised, double-blind, parallel group, single-center study with an intervention period of four weeks. The study was conducted at CRST (Clinical Research Services of the University of Turku) following the principles of Good Clinical Practise.

**Figure 1:**
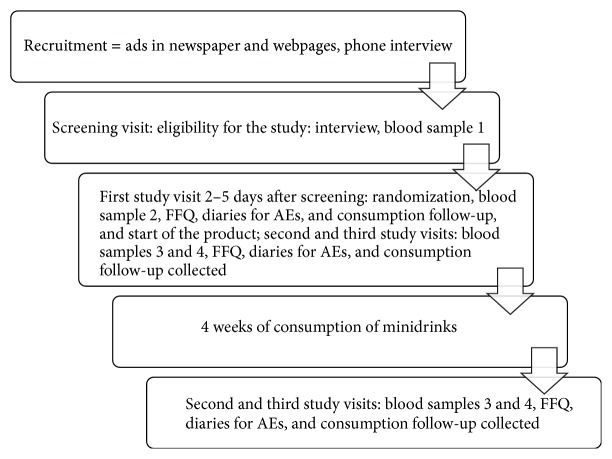
Study design.

The study included four visits to the study unit including a screening visit. The study design is illustrated in Figure 1. On the screening visit, the subjects met the study physician and were further informed in detail about the study, its protocol, and the study products. Thereafter, fasting blood was sampled for analyses of serum total cholesterol, HDL cholesterol and triacylglycerol concentrations, *µ*CRP, blood glucose, and thyroid function. Blood pressure was measured three times (of which the average was calculated) and height and body weight were determined. Furthermore, subjects had to complete a medical and general questionnaire and were given a food frequency questionnaire to be filled at home.

Those subjects who met the inclusion and none of the exclusion criteria at screening came to the first study visit 3–5 days after the screening visit. During the first study visit, subjects were randomized to one of the study groups and fasting blood samples for serum lipids and *µ*CRP were taken. The subjects received the study products as well as the diaries to be filled daily on product consumption details and possible symptoms experienced during the days of the study. Subjects were advised to follow their habitual diet and to keep their lifestyle unaltered during the study. The second and third study visits were 3 days apart during the last week of the study and were identical to the first study visit. During the last visit, the subjects returned the diaries and the products they had not used during the trial, if any.

### 2.3. Test Products and Diet

The subjects were advised to consume one test drink daily with a main meal, preferably with lunch, for four weeks. The nutrient contents of the test drinks (65 mL) are given in Table 1. The drinks were otherwise identical but contained differing amounts of plant stanols (0 g, 1.6 g, or 2.0 g) as fatty acid esters and camelina oil (0 g or 2.0 g). The analyzed content of plant stanols was 0 g, 1.5 g, or 1.9 g and the analysed content of plant stanols + plant sterols 0 g, 1.6 g, or 2.0 g (Table 1). Of the plant stanols, 89% was sitostanol and 11% campestanol.

The subjects were advised to take the yoghurt drink with a main meal of the day. To ensure and check compliance, the subjects were asked to record details of their use of the test drinks (date, precise clock time, and the food that was consumed together with the drink) in a study diary every day for the 4 weeks of the trial. They were also asked to fill in any failures to take the product and also if they did not consume the total contents of the yoghurt drink.

The subjects were advised to keep their diet unaltered during the study, that is, not to make any major changes in their way of eating or choices of foods. To check for possible changes in diets during the 4-week intervention period, the subjects were asked to fill in a food frequency questionnaire at the beginning and at the end of the study to record the consumption of the most relevant foods which may influence serum cholesterol levels. The food frequency questionnaire was checked immediately at the study visits in the presence of the subjects by study personnel for incomplete filling, major changes in diet, or other deviations.

The Ethics Committee of the University of Turku approved the protocol and all subjects signed an informed consent.

### 2.4. Blood Sampling and Analysis of Serum Lipids

Fasting blood samples were taken by venipuncture twice at the beginning of the study (at screening and first study visit) and twice at the end of the study (3 days apart, i.e., at the second and third study visits). Subjects were not allowed to eat after 20.00 h the day preceding blood sampling, smoke in the morning of the blood sampling, and use alcohol 24 h before blood sampling. Laboratory samples were analyzed with routine standardized methods at Turku University Central Hospital. Serum total and HDL cholesterol and triglycerides were measured using standard enzymatic methods and LDL cholesterol concentration was calculated using the Friedewald formula [29].

For serum lipids, baseline values were evaluated as a mean of measurements at the screening and at the first visit. Correspondingly, values at the end of the study were evaluated as a mean of measurements at the second and at the third visit. This procedure was used to reduce the effect of day-by-day variability in serum cholesterol on results.

### 2.5. Statistical Analyses

Based on a power calculation in regard to assumed LDL cholesterol difference between the placebo group and the plant stanol groups, a sample size was determined to be 30 subjects per group, already taking into account few possible dropouts. As the number of the interested participants was larger, a total of 155 subjects (31/group) were recruited. The subjects were randomised stratified by gender and by serum levels of total cholesterol to ensure the comparability of the treatment groups, into four groups each receiving one of the plant stanol ester products and a placebo group receiving a product with no added plant stanol.

Primary efficacy variable in this study was the percent change from baseline in serum LDL cholesterol. Secondary efficacy variables were the absolute change from baseline in serum LDL cholesterol and the absolute and percent change from baseline in other lipids, that is, serum total cholesterol, HDL cholesterol, triglycerides, and variables derived from laboratory measurements (non-HDL cholesterol, HDL/cholesterol ratio, HDL/non-HDL ratio, and HDL/LDL ratio), as well as *µ*CRP.

The statistical analyses were performed on the individual data. Continuous demographic and baseline variables (age, height, waist, weight, BMI, and blood pressure) were analysed using a one-way ANOVA model between the treatment groups. Distributions of categorical variables (gender, consumption pattern) between treatment groups were analysed using a chi-square test. Analyses for primary and secondary efficacy variables were done for the absolute and percent change from baseline to the end-of-study evaluation. Treatment differences were evaluated using the one-way ANOVA model with treatment as a main effect. Furthermore, the magnitude of the treatment differences in pairwise comparisons was evaluated by constructing estimates and their 95% confidence intervals for the differences. Changes from baseline within groups for the primary and secondary efficacy variables were analysed by Wilcoxon signed ranks test. All statistical analyses were done using IBM SPSS Statistics Version 23 (IBM Corp.). The results are presented as means ± SD and with 95% confidence intervals (percent change as compared to the placebo group). In the statistical analyses a *p* value less than 0.05 was considered as statistically significant.

## 3. Results

### 3.1. Subject Characteristics and Compliance

Of the 155 subjects that were recruited, 7 subjects withdrew from the study before the start of the intervention because of personal reasons. Baseline characteristics of the remaining 147 subjects that completed the study are presented in Table 2. There were no significant differences between the groups in gender, age, waist, BMI, systolic and diastolic blood pressure, CRP, or serum lipid levels measured at the screening visit.

The subjects kept a study diary, where they recorded the use of the test drink, as well as the food that was consumed together with the drink on every day of the intervention. The study diaries revealed that three subjects had finished the consumption of the test product several days before the end of the study. These subjects were removed from the final analyses. One subject had participated in a marathon race during the intervention period, and also her results were excluded from the final analyses. Thus 143 subjects were included in the statistical analyses.

Of the 143 subjects who were included in the statistical analyses, 133 (93%) reported consumption of the test drink together with a main meal (lunch, dinner, and breakfast), mostly with lunch, as advised (Table 3). Six subjects (4%) consumed the drink mainly with snacks or on few days on its own, without other foods. Four subjects (3%) failed to return their study diaries. There were no statistically significant differences between the groups in regard to the consumption pattern. The statistical analyses of the primary and secondary efficacy variables were rerun without these ten subjects, but the main results remained essentially the same. The overall self-reported consumption rate of the drinks was 99.5% of the provided bottles among the 139 subjects who returned their diaries.

### 3.2. Serum Lipids

All treated groups showed statistically significant lowering of serum total, LDL, and non-HDL cholesterol at the end-of-study measurement relative to the baseline measurement (Table 4). The placebo group did not indicate any cholesterol lowering effect. Compared to the placebo group, the LDL cholesterol was reduced by 0.35 mmol/L or 9.4% (*p* < 0.01) and 0.31 mmol/L or 8.1% (*p* < 0.01) in the groups receiving 1.6 g plant stanols and 2.0 g plant stanols, respectively. The group receiving 1.6 g plant stanols and camelina oil showed 0.39 mmol/L or 11.0% (*p* < 0.01) and the group receiving 2.0 g plant stanols and camelina oil 0.33 mmol/L or 8.4% (*p* < 0.01) reduction in LDL cholesterol compared to the placebo group. There were no significant differences between the treated groups in the total, LDL, or non-HDL cholesterol change. The statistics for the baseline and the end-of-study values as well as those for absolute and percentual changes are given in Table 4.

In serum triglycerides, there were no statistically significant differences between the end-of-study measurement and the baseline measurement in any of the groups. No significant differences were found between the treated groups and placebo or between the treated groups, either (Table 4). HDL cholesterol was reduced in the 1.6 g plant stanol + camelina group compared to baseline, but not as compared to placebo (Table 4). The ratios of HDL cholesterol to total cholesterol (HDL/total cholesterol), to non-HDL cholesterol (HDL/non-HDL), and to LDL cholesterol (HDL/LDL) were increased statistically significantly in all treated groups compared to the placebo group (Table 4). No significant differences were found between the groups in *µ*CRP.

The influence of camelina oil was evaluated in the context of primary and secondary analyses. Results showed that there were no statistically significant differences in any primary or secondary variable between the subjects treated with plant stanols only and subjects treated with plant stanols and camelina oil (Table 4).

## 4. Discussion

The current study investigated the effects of plant stanol ester either with or without added vegetable oil on the serum cholesterol levels in moderately hypercholesterolemic subjects, when administered in a yoghurt minidrink together with a meal. The aim was to evaluate whether addition of a small amount of vegetable oil would promote the cholesterol lowering effect of plant stanol ester. Coemulsification of camelina oil with plant stanol ester into the minidrink could potentially enhance the cholesterol lowering effect of such a product by several mechanisms.

In our trial, all four plant stanol ester products equally reduced total and LDL cholesterol compared to baseline and compared to placebo. The mean LDL cholesterol was lowered by 0.35 mmol/L (9.4%) with 1.6 grams of plant stanols and by 0.31 mmol/L (8.1%) with 2.0 grams compared to placebo. When a dose of 2 grams of camelina oil was added, LDL cholesterol was reduced slightly further, by 0.39 mmol/L (11.0%) and 0.33 mmol/L (8.4%) with 1.6 g and 2.0 g plant stanol products, respectively. A surprising finding was the slight numerically enhanced reduction in serum LDL cholesterol with the smaller daily dose of 1.6 g of plant stanols than with 2.0 g both with and without added camelina oil. However, none of the differences between plant stanol ester products were statistically significant.

Triggering the physiological responses to food intake is crucial for the optimal efficacy of plant stanol and plant sterol food products [23, 24]. Plant stanols and plant sterols reduce circulating LDL cholesterol level by reducing the absorption of dietary and biliary cholesterol from the small intestine, the main mechanism suggested to be through competitively inhibiting cholesterol incorporation in the mixed micelles of bile salts [30]. Thus gallbladder contraction and subsequent bile secretion, as well as simultaneous delivery of pancreatic enzymes including cholesterol esterase and plant stanol ester or plant sterol ester into the upper part of the duodenum, are necessary for the cholesterol lowering efficacy of plant stanols and plant sterols [23, 24]. Bile secretion into the duodenum is regulated by hormone cholecystokinin (CCK), which is released into intestine in response to macronutrients, especially fat [23]. It has been shown that a low-fat yogurt minidrink containing plant sterol ester does not sufficiently trigger gallbladder contraction and bile flow to exhibit cholesterol lowering efficacy if consumed on its own without coingestion of a solid meal [24]. Fat is the strongest stimulus for gallbladder contraction, and a threshold level of 2 g fat has been suggested [9]. Marciani et al. showed that the fat content of a plant stanol ester beverage correlated with the maximum gallbladder volume change after ingestion of the beverages without a concurrent meal [23]. Also the postprandial plasma CCK concentration was dependent on the fat content of the beverage, beverages containing 6.5 g or 10 g fat yielding significantly higher CCK response than a beverage containing 1.5 g fat [23].

In our study, the minidrink was advised to be consumed together with a meal, preferably lunch. A typical Finnish lunch contains enough fat to induce sufficient CCK response, gallbladder contraction, and bile flow for plant stanol ester to exhibit its cholesterol lowering efficacy. However, a minidrink product is easily adopted as a snack-type concept by consumers. Therefore, such a product may be consumed on its own without a sufficient meal despite the guidance provided in label of the product. The challenges with this kind of a minidrink concept were previously demonstrated in at least two clinical trials. Doornbos et al. showed with a plant sterol ester minidrink that the drink was much more efficient when ingested together with a meal (9.5% reduction in LDL cholesterol) than when ingested before breakfast on an empty stomach (5.1% reduction in LDL cholesterol) [9]. Also, Seppo et al. [25] hypothesized that the poor LDL cholesterol lowering (3.2% reduction in LDL) recorded in one arm of their dairy product trial with plant stanol ester was because the study subjects may have consumed the product on an empty stomach in spite of the advice given. When the same product was consumed in a more controlled setting after lunch, LDL cholesterol was effectively reduced by 11.8%. We hypothesized that, by adding some vegetable oil to the minidrink, we could enhance the cholesterol lowering efficacy. In our study, the subjects kept a diary of the minidrink consumption recording the food that was taken together with the minidrink. The diaries revealed that in this study majority (93%) of the subjects consumed the minidrinks together with a main meal as advised. The statistical analyses were rerun without the ten subjects, who consumed the minidrink mainly with snacks or who failed to return their diaries. However, this did not have any major impact on the results and all plant stanol ester yoghurt drinks lowered cholesterol equally effectively when compared to the placebo drink (results not shown). The calorie or fat content of the meals was not analysed so the effect of these parameters cannot be estimated in this study.

In food formulations, plant stanols and plant sterols are typically esterified with vegetable oil fatty acids for better lipid solubility. For optimal cholesterol reducing effect, however, the ester bond must be effectively hydrolysed in the duodenum [22, 30]. The hydrolysis is accomplished with pancreatic cholesterol esterase released in the duodenum after stimulus of food entering the stomach and the duodenum. Thus not only the gallbladder contraction and bile flow but also simultaneous pancreatic enzyme secretion is a prerequisite for the cholesterol lowering efficacy of plant stanol ester. Earlier research has estimated the extent of plant stanol ester hydrolysis to be close to 90% [31, 32]. The fat content of the concurrent meal has an effect on the hydrolysis rate of plant stanol ester. In a crossover trial, only 40% of plant stanol ester was hydrolysed when a fat-free plant stanol ester product was consumed together with a low-fat diet [20], compared to a hydrolysis rate of 70% when ingested with a normal fat diet. The rate of plant stanol or plant sterol ester hydrolysis by cholesterol esterase has been found to be dependent on both the stanol/sterol and the fatty acid moieties* in vitro*, and addition of free fatty acids into the reaction mixture increased the enzyme activity [30]. Recently, Lubinus et al. showed with minidrinks that there is a large interindividual variability in the extent of hydrolysis of plant stanol ester and plant sterol ester when evaluated using content of unhydrolysed plant stanol or sterol esters in the faeces. They also showed that the fatty acid moiety has a significant impact on hydrolysis rate of plant stanol/sterol esters [28]. Oleate, linoleate, and linolenate presented the most effective hydrolysis rates, whereas eicosanoate and palmitate were less easily hydrolysed. Although the influence of the plant sterol/stanol moiety was less pronounced in the study of Lubinus et al., there was a (nonsignificant) trend for lower average hydrolysis rate for plant stanol esters as compared to plant sterol esters. However, in the study of Lubinus et al. the minidrinks were not advised to be taken together with meals. This may have influenced the results and increased the interindividual variation recorded. We hypothesized that adding camelina oil to the minidrinks may enhance the cholesterol lowering efficacy by ensuring more effective incorporation of the plant stanol ester into the fat phase in the stomach and by increasing the rate and extent of hydrolysis of the ester bond and thus make plant stanols more easily available for micellar incorporation.

The microstructure of fat emulsion affects its digestion [26, 27]. The stability of the emulsion in the acidic gastric conditions has been reported to affect its gastric emptying rate and subsequent lipolysis [33]. Acid stable emulsions exhibit steady and slower gastric emptying patterns, increased gallbladder contraction, and increased postprandial plasma CCK levels compared to acid unstable emulsions [33]. Recently Steingoetter et al. showed that not only the acid stability but also the solid fat content of the fat phase affects the gastric behaviour and subsequent digestion of triglyceride emulsions [27]. An acid unstable emulsion in which 30% of the fat was solid separated into liquid oil and solid fat aggregates in stomach. The solid fat aggregates were resistant to reemulsification by gastric movements and had low digestibility. Faster gastric emptying and reduced gallbladder contraction were observed after the ingestion of this emulsion. Remarkably, no increase at all in blood triglycerides was recorded although 70% of the fat was liquid rapeseed oil. On the contrary, an acid unstable emulsion with liquid fat (rapeseed oil) was redispersed and reemulsified in stomach more easily after the initial separation and had slower gastric emptying than the emulsion with solid fat. The emulsion consisting only of liquid oil showed similar gallbladder contraction and triglyceride absorption pattern to acid stable emulsions [27]. The solid fat content (SFC) of plant stanol ester with the plant stanols esterified with rapeseed oil fatty acids is 10–15% at 35°C and 5–8% at 40°C. Plant stanol ester minidrinks are typically stored refrigerated. In refrigerated minidrink a larger proportion of plant stanol ester or plant sterol ester is in solid form, which may impact on the incorporation of the plant sterol or stanol ester into the emulsified fat in the chyme. Blending camelina oil and plant stanol ester before emulsification into the milk matrix might help in the incorporation of plant stanol ester into the fat phase of the chyme and promote more effective emulsification with a more even distribution of plant stanol ester throughout the emulsified fat in the stomach. We hypothesized that this could enhance the cholesterol lowering efficacy of the plant stanol ester yoghurt drinks.

Our trial was a clinical study instead of a mechanistic evaluation and could only provide evidence to support the working hypotheses of improving the cholesterol lowering efficacy of plant stanol ester minidrinks by addition of a vegetable oil. Therefore, we can only compare the LDL reduction between the groups with and without camelina oil. In our study, adding 2 g camelina oil to the yoghurt minidrinks did not enhance the LDL cholesterol lowering efficacy of plant stanol ester. Although in the 1.6 g group there was a numerically slightly higher reduction in LDL cholesterol when camelina oil was added (11.0% versus 9.4%), the differences were not statistically significant. The result is in accordance with the study of Doornbos et al. [9], where yoghurt minidrinks containing plant sterol ester and either 0.1 g or 1.5 g dairy fat had similar cholesterol lowering efficacy when consumed with the lunch. The typical mean fat intake with the lunch ranged from 7 to 42 g and had no effect on the magnitude of LDL reduction [9]. In our study, the more solid plant stanol ester was used instead of plant sterol ester, the fat was liquid vegetable oil rather than dairy fat, and the fat content of the minidrinks is somewhat higher than in the study of Doornbos et al. [9], all of which could have contributed favorably to an enhanced cholesterol lowering effect. However, these differences did not result in an enhanced cholesterol lowering effect of the studied minidrinks. The minidrinks were consumed together with meals. Possibly the effects of camelina oil on the cholesterol lowering of plant stanol ester minidrinks would have been different if the minidrinks had been advised to be taken together with snacks or without other foods.

In addition to acting as a carrier for plant stanols and plant sterols, the food matrix can further promote the LDL cholesterol lowering capacity through its fatty acid composition. Substituting saturated fat or carbohydrates with monounsaturated and especially polyunsaturated fatty acids is known to lower cholesterol independent of plant stanols and plant sterols. Camelina oil is rich in polyunsaturated fatty acids, especially alpha-linolenic acid, omega-3 polyunsaturated fatty acid. The alpha-linolenic acid content of camelina oil is typically 34%. Thereby, addition of camelina oil improves the overall fatty acid composition of the diet, and it has been shown to lower cholesterol in a comparable manner to rapeseed oil [34]. Long chain omega-3 fatty acids eicosapentaenoic acid (EPA) and docosahexaenoic acid (DHA) lower serum triglycerides, also when consumed together with plant sterols [11, 35, 36]. The combination of plant sterols and EPA and DHA has also been suggested to enhance the cholesterol lowering effect of plant sterols [35]. The effects of alpha-linolenic acid on serum triglycerides are more controversial, and in most studies no triglyceride lowering has been found [37]. In our study, addition of 2 g alpha-linolenic acid-rich camelina oil did not change the serum triglyceride levels.

In the present study, LDL cholesterol was lowered numerically slightly more with the smaller dose of 1.6 g of plant stanols than with 2.0 g, both with and without added camelina oil, although the differences were not statistically significant. Dose-response effect has been previously shown with plant stanol and plant sterol products [5, 6]. The degree of LDL cholesterol lowering found in our study with a low-fat minidrink format falls closely within the range predicted by several meta-analyses incorporating data from studies with a wide range of different food vehicles used as carriers for plant stanols and plant sterols. The early meta-analyses were done based on studies where plant stanols were added mainly in margarines [2, 3]. More recent meta-analyses have incorporated data from a wider range of food formats, ranging from margarines and mayonnaises to yogurt, milk, cheese, meat, grain, juice, and salad dressings and even chocolate. AbuMweis et al. [4] approximated an LDL cholesterol lowering effect 0.32 mmol/L in adults of a similar age range as ours and a 0.29 mmol/L reduction with a sterol/stanol dose range between 1.5 and 2.0 grams. When the carrier food was milk or yoghurt, the estimated LDL cholesterol reduction was 0.34 mmol/L in AbuMweis et al.'s analysis. They also calculated the LDL cholesterol lowering effect based on frequency and time of intake of the sterol/stanol products and arrived at an estimate of only 0.14 mmol/L when the products were ingested once a day in the morning but 0.30 mmol/L when the product was consumed once a day in the afternoon or with the main meal as also advised in our study. According to the meta-analysis by Musa-Veloso et al. [5] LDL cholesterol would be lowered by 7.7% with 1.6 grams/day of plant stanols and by 9.1% with 2 grams per day based on data of all available food formats (weighted analysis, no dose restriction) [5]. Recently, the meta-analysis by Ras et al. [6] estimated an LDL reduction of 6.7% with plant stanol dose between 1.5 and 2 grams and 10% with a dose between 2.0 and 2.5 grams.

The vast majority of data on plant stanol ester dose effects on LDL cholesterol has focussed on doses of 2 grams and above, and 1.6 g has been addressed only in a few studies. Woodgate et al. [38] studied the effect of plant stanol ester in capsules and found that 1.6 grams of plant stanol reduced LDL by 9% when ingested in two daily doses, 3 capsules with breakfast and 3 capsules with dinner. Another study with 1.6 g dose was a dose-response study where 5 doses of plant stanols (0, 0.8, 1.6, 2.4, and 3.2 g/d) incorporated in margarines were tested [39]. They found 5.6% (0.27 mmol/L) and 9.7% (0.47 mmol/L) reductions with 1.6 g and 2.4 grams, respectively. The daily doses were consumed in 25 grams of vegetable oil based margarine (70% fat) and were divided into two to three doses per day. Baseline LDL cholesterol was markedly higher than in our trial, namely, 4.81 mmol/L. Other dose-range studies have evaluated effects of doses up to 9 grams of plant stanol and have shown a continuous dose-response effect [40, 41].

Early trials used fat-based food vehicles because of better solubility of plant stanols and plant sterols in such matrices. The first food products available for commercial use were therefore also fat-based products, that is, margarines. Most of the more recent applications are different formats of dairy products such as yoghurts or dairy minidrinks. With 2 g plant stanols (as plant stanol ester) in minidrinks, previously LDL cholesterol lowering of 0.34–0.37 mmol/L or 8–10% has been demonstrated [13, 15, 18]. Our results are well in accordance with these studies. Seppo et al. [25] reported data on two trials with a 100 mL dairy minidrink. They found a widely varying response in LDL cholesterol depending on whether the test product was consumed under supervision with a meal or just advised to be taken with a meal (LDL cholesterol reduction by 11.8% or 3.2%, resp.). Although plant stanol dose of 1.6 g has not previously been studied in a yoghurt minidrink format, studies with 1.6 g plant sterols in this product format exist. Trials with 1.6 g dose of plant sterols (as plant sterol ester) have shown a range of LDL cholesterol lowering between 0.35 and 0.49 mmol/L or from 8.3% to 12.2% [10, 12, 42]. In our trial, the dose of 1.6 g plant stanols resulted in 9.4% LDL cholesterol lowering, which is comparable to these plant sterol results. Two other studies with 1.6 g plant sterols and with a bigger volume of the drink demonstrated a 12.4% [43] and a 5.2% [44] reduction in LDL cholesterol compared to placebo. With 2 g plant sterols in minidrinks, LDL cholesterol reduction of 4.5% [11] has been reported. In larger volume drinks, 2 g plant sterols have reduced LDL cholesterol by 6–8% [45] or 4.1% [46]. Interestingly, 1.6 g daily dose of plant sterols as plant sterol ester seems to give a numerically better LDL reduction than the 2 g dose in minidrinks [10–12, 42] although these doses were not assessed in the same trial. In our study, the differences in LDL cholesterol reduction between 1.6 g and 2 g plant stanols, both with and without camelina oil addition, were not statistically significant, although there seemed to be numerically higher LDL reduction in the 1.6 g groups. Whether the numerically higher reduction in LDL cholesterol in the 1.6 g groups was just a chance finding or whether, for example, the product format affected the results, remains to be elucidated in further studies.

## 5. Conclusions

This study showed that 1.6 g or 2 g plant stanols provided as plant stanol ester in yoghurt minidrinks are effective in lowering serum total, LDL, and non-HDL cholesterol when consumed once a day with a meal. Addition of a small amount (2 g) of camelina oil did not enhance the cholesterol lowering effects of plant stanol ester. The omega-3-rich camelina oil did not affect the serum triglyceride levels either. This is the first study demonstrating the cholesterol lowering efficacy of 1.6 g plant stanols in the minidrink product format. This is also the first study to investigate the combined effects of plant stanols and an omega-3-rich vegetable oil in a minidrink format on serum lipids.

## Figures and Tables

**Table 1:** Nutritional values of the yoghurt drinks.

Yoghurt drink	Placebo	1.6 g stanols	2 g stanols	1.6 g stanols + camelina^1^	2 g stanols + camelina^1^
	Per 100 g/65 mL				
Energy (kJ)	376/244	339/220	349/227	445/289	463/301
Energy (kcal)	90/58	81/53	83/54	106/69	111/72
Protein (g)	3.5/2.3	3.4/2.2	3.4/2.2	3.3/2.2	3.3/2.1
Carbohydrates (g)	13/8.7	13/8.5	13/8.5	13/8.3	13/8.2
Fat^2^ (g)	2.5/1.6	1.7/1.1	2.0/1.3	4.7/3.0	5.2/3.4
Polyunsaturated fat^2^ (g)	0.1/0.0	0.5/0.3	0.6/0.4	2.2/1.4	2.2/1.5
Alpha-linolenic acid^2^ (g)	0.0/0.0	0.1/0.1	0.2/0.1	1.2/0.8	1.2/0.8
Monounsaturated fat^2^ (g)	0.2/0.1	1.0/0.7	1.2/0.8	2.1/1.3	2.3/1.5
Saturated fat^2^ (g)	2.2/1.5	0.2/0.1	0.2/0.1	0.5/0.3	0.6/0.4
Trans fat (g)	0.0/0.0	0.0/0.0	0.0/0.0	0.0/0.0	0.1/0.1
Fiber (g)	0.0/0.0	0.0/0.0	0.0/0.0	0.0/0.0	0.0/0.0
Plant stanols (g)	0.0/0.0	2.4/1.5	3.0/1.9	2.3/1.5	3.0/1.9
Plant stanols + sterols (g)	0.0/0.0	2.4/1.6	3.0/2.0	2.4/1.6	3.1/2.0

^1^Typical fatty acid composition of camelina oil: 54% polyunsaturated fatty acids (34% alpha-linolenic acid), 33% monounsaturated fatty acids, 11% saturated fatty acids, and 2% trans fatty acids.

^2^Including the fatty acids derived from plant stanol ester.

**Table 2:** Baseline characteristics of the subjects^1,2^.

	Group				
	Placebo	1.6 g	2 g	1.6 g + camelina	2 g + camelina
	*N* = 31	*N* = 28	*N* = 29	*N* = 29	*N* = 30
Gender					
Male	8	16	10	13	15
Female	23	12	19	16	15
Age (y)	49 ± 9.5	47.6 ± 11.6	49.4 ± 9.1	47.1 ± 9.5	49.0 ± 10.4
BMI (kg/m^2^)	25.1 ± 2.9	24.7 ± 3.1	24.7 ± 3.0	25.6 ± 2.6	24.7 ± 3.1
Waist (cm)					
Male	92.5 ± 7.6	95.3 ± 8.2	91.7 ± 8.9	96.0 ± 6.3	92.8 ± 5.9
Female	83.1 ± 10.4	78.9 ± 7.4	82.7 ± 8.7	84.1 ± 9.6	82.1 ± 9.9
BP_syst_ (mmHg)	128 ± 18.6	133 ± 19.1	126 ± 17.2	127 ± 14.6	129 ± 15.1
BP_diast_ (mmHg)	83 ± 99	81 ± 10.7	81 ± 11	83 ± 9.8	82 ± 9.9
Total cholesterol (mmol/L)	6.32 ± 0.65	5.97 ± 0.64	6.13 ± 0.74	6.07 ± 0.78	6.08 ± 0.78
LDL cholesterol (mmol/L)	3.89 ± 0.83	3.56 ± 0.65	3.76 ± 0.74	3.59 ± 0.76	3.77 ± 0.68
HDL cholesterol (mmol/L)	1.87 ± 0.47	1.86 ± 0.42	1.84 ± 0.49	1.90 ± 0.55	1.67 ± 0.45
Triacylglycerol (mmol/L)	1.26 ± 0.47	1.23 ± 0.62	1.19 ± 0.43	1.28 ± 0.61	1.40 ± 0.63
Non-HDL cholesterol (mmol/L)	4.44 ± 0.89	4.12 ± 0.77	4.29 ± 0.77	4.17 ± 0.94	4.40 ± 0.83
HDL/total cholesterol ratio	0.30 ± 0.09	0.31 ± 0.08	0.30 ± 0.07	0.32 ± 0.10	0.28 ± 0.08
CRP (mg/L)	1.62 ± 1.73	1.79 ± 3.11	1.07 ± 1.20	1.84 ± 2.08	1.06 ± 0.77
Glucose (mmol/L)	5.26 ± 0.48	5.52 ± 0.49	5.35 ± 0.32	5.29 ± 0.45	5.44 ± 0.41
TSH (mU/L)	2.08 ± 1.03	2.09 ± 0.94	2.05 ± 0.91	2.14 ± 1.14	1.92 ± 1.14

^1^Differences between groups were analysed by ANOVA and chi-square tests and were not statistically significant.

^2^Mean ± SD.

**Table 3:** Consumption pattern of the yoghurt drinks. Number of subjects taking the drink with main meals, with snacks, or subjects who failed to return the study diary.

	Group				
	Placebo	1.6 g	2 g	1.6 g + camelina	2 g + camelina
	*n* = 30	*n* = 28	*n* = 27	*n* = 29	*n* = 29
With main meal	30	27	25	26	25
With snacks	0	0	2	1	3
Failed to return the diary	0	1	0	2	1

**Table 4:** Effects of plant stanol yoghurt minidrinks with or without added camelina oil on serum lipids^1^.

	Group					
	Placebo	1.6 g	2 g	1.6 g + camelina	2 g + camelina	*p* value^2^
	*n* = 30	*n* = 28	*n* = 27	*n* = 29	*n* = 29	
Total cholesterol						
Baseline	6.35 ± 0.64	5.97 ± 0.64	6.12 ± 0.77	6.07 ± 0.78	6.11 ± 0.77	NS
End-of-study	6.38 ± 0.83	5.64 ± 0.58^b^	5.84 ± 0.79^b^	5.68 ± 0.81^b^	5.81 ± 0.76^b^	0.002
Absolute change from baseline	0.03 ± 0.47	−0.34 ± 0.52	−0.28 ± 0.42	−0.39 ± 0.42	−0.30 ± 0.49	0.008
% change from baseline	0.31 ± 7.53	−5.23 ± 8.38	−4.50 ± 6.70	−6.34 ± 6.70	−4.72 ± 8.02	0.010
% change from placebo		−5.54^*∗∗*^	−4.80^*∗*^	−6.65^*∗∗*^	−5.03^*∗*^	
95% CI		−9.44 to −1.64	−8.74 to −0.87	−10.51 to −2.78	−8.89 to −1.17	
LDL cholesterol						
Baseline	3.93 ± 0.80	3.56 ± 0.65	3.77 ± 0.76	3.59 ± 0.76	3.80 ± 0.67	NS
End-of-study	3.99 ± 0.87	3.27 ± 0.61^b^	3.53 ± 0.74^b^	3.26 ± 0.77^b^	3.53 ± 0.65^b^	0.001
Absolute change from baseline	0.06 ± 0.40	−0.29 ± 0.40	−0.25 ± 0.35	−0.33 ± 0.40	−0.27 ± 0.43	0.001
% change from baseline	1.79 ± 10.51	−7.62 ± 10.23	−6.30 ± 9.42	−9.22 ± 10.94	−6.62 ± 11.39	0.001
% change from placebo		−9.42^*∗∗*^	−8.09^*∗∗*^	−11.02^*∗∗*^	−8.42^*∗∗*^	
95% CI		−14.89 to −3.94	−13.62 to −2.56	−16.45 to −5.60	−13.84 to −2.99	
HDL cholesterol						
Baseline	1.85 ± 0.46	1.86 ± 0.42	1.82 ± 0.49	1.90 ± 0.55	1.67 ± 0.46	NS
End-of-study	1.81 ± 0.48	1.81 ± 0.43	1.78 ± 0.48	1.82 ± 0.57^b^	1.66 ± 0.41	NS
Absolute change from baseline	−0.04 ± 0.14	−0.05 ± 0.15	−0.04 ± 0.16	−0.08 ± 0.15	0.01 ± 0.15	NS
% change from baseline	−2.50 ± 7.19	−2.38 ± 8.06	−2.02 ± 7.64	−4.21 ± 8.42	0.62 ± 10.13	NS
% change from placebo		0.13	0.46	−1.71	3.12	
95% CI		−4.21 to 4.47	−3.91 to 4.84	−6.01 to 2.60	−1.18 to 7.42	
Non-HDL cholesterol						
Baseline	4.50 ± 0.85	4.12 ± 0.77	4.30 ± 0.79	4.17 ± 0.94	4.44 ± 0.82	NS
End-of-study	4.57 ± 0.93	3.83 ± 0.75^b^	4.06 ± 0.78^b^	3.86 ± 0.94^b^	4.15 ± 0.81^b^	0.007
Absolute change from baseline	0.07 ± 0.43	−0.29 ± 0.45	−0.24 ± 0.35	−0.31 ± 0.40	−0.29 ± 0.47	0.003
% change from baseline	1.78 ± 10.17	−6.64 ± 10.12	−5.40 ± 8.15	−7.38 ± 9.41	−6.31 ± 10.99	0.003
% change from placebo		−8.41^*∗∗*^	−7.17^*∗∗*^	−9.16^*∗∗*^	−8.09^*∗∗*^	
95% CI		−13.52 to −3.30	−12.33 to −2.01	−14.22 to −4.09	−13.15 to −3.02	
Triacylglycerol						
Baseline	1.27 ± 0.48	1.23 ± 0.62	1.17 ± 0.43	1.28 ± 0.61	1.41 ± 0.64	NS
End-of-study	1.29 ± 0.39	1.23 ± 0.63	1.18 ± 0.37	1.36 ± 0.77	1.39 ± 0.71	NS
Absolute change from baseline	0.02 ± 0.34	−0.01 ± 0.53	0.01 ± 0.25	0.08 ± 0.36	−0.02 ± 0.40	NS
% change from baseline	6.99 ± 26.97	2.07 ± 31.39	5.14 ± 25.79	6.57 ± 26.16	0.96 ± 26.95	NS
% change from placebo		−4.92	−1.85	−0.42	−6.02	
95% CI		−19.22 to 9.38	−16.28 to 12.58	−14.59 to 13.75	−20.19 to 8.14	
HDL/total cholesterol						
Baseline	0.30 ± 0.08	0.31 ± 0.08	0.30 ± 0.08	0.32 ± 0.10	0.28 ± 0.08	NS
End-of-study	0.29 ± 0.08^a^	0.33 ± 0.09^a^	0.31 ± 0.08	0.33 ± 0.10	0.29 ± 0.08^a^	NS
Absolute change from baseline	−0.01 ± 0.02	0.01 ± 0.02	0.01 ± 0.02	0.01 ± 0.03	0.01 ± 0.03	0.017
% change from baseline	−2.48 ± 8.48	3.35 ± 7.76	2.72 ± 7.16	2.55 ± 9.86	6.11 ± 11.73	0.011
% change from placebo		5.82^*∗*^	5.19^*∗*^	5.02^*∗*^	8.58^*∗∗*^	
95% CI		1.05 to 10.59	0.38 to 10.01	0.30 to 9.75	3.86 to 13.31	
HDL/non-HDL cholesterol						
Baseline	0.44 ± 0.18	0.48 ± 0.18	0.45 ± 0.17	0.50 ± 0.24	0.40 ± 0.15	NS
End-of-study	0.42 ± 0.16^a^	0.51 ± 0.21^a^	0.46 ± 0.16	0.52 ± 0.26	0.43 ± 0.16^a^	NS
Absolute change from baseline	−0.02 ± 0.06	0.03 ± 0.06	0.02 ± 0.05	0.02 ± 0.08	0.03 ± 0.07	0.021
% change from baseline	−3.45 ± 12.12	5.51 ± 11.68	4.04 ± 10.51	4.40 ± 15.21	8.84 ± 16.99	0.013
% change from placebo		8.96^*∗*^	7.49^*∗*^	7.85^*∗*^	12.29^*∗∗*^	
95% CI		1.91 to 16.01	0.38 to 14.60	0.87 to 14.83	5.31 to 19.28	
HDL/LDL						
Baseline	0.51 ± 0.22	0.55 ± 0.19	0.51 ± 0.18	0.58 ± 0.28	0.46 ± 0.16	NS
End-of-study	0.49 ± 0.20^a^	0.59 ± 0.22^a^	0.53 ± 0.19	0.63 ± 0.31	0.49 ± 0.17^a^	NS
Absolute change from baseline	−0.02 ± 0.06	0.04 ± 0.06	0.02 ± 0.06	0.04 ± 0.09	0.03 ± 0.08	0.005
% change from baseline	−3.48 ± 11.84	6.34 ± 10.75	5.23 ± 11.66	7.71 ± 15.29	9.21 ± 15.87	0.004
% change from placebo		9.83^*∗∗*^	8.71^*∗*^	10.49^*∗∗*^	12.70^*∗∗*^	
95% CI		2.91 to 16.75	1.73 to 15.71	3.63 to 17.36	5.84 to 19.56	

^1^Values are means ± SD.

^2^Between groups.

NS: statistically nonsignificant.

^a^ *p* < 0.05 significantly different from baseline. ^b^ *p* < 0.01 significantly different from baseline.

^*∗*^ *p* < 0.05 significantly different from placebo; ^*∗∗*^ *p* < 0.01 significantly different from placebo.
